# A Universal Solution of Controlling the Distribution of Multimaterials during Macroscopic Manipulation via a Microtopography-Guided Substrate

**DOI:** 10.3390/nano8121036

**Published:** 2018-12-12

**Authors:** Changhai Li, Fengqiang Zhang, Jia Zhang, Bin Guo, Zhenlong Wang

**Affiliations:** 1 Academy of Fundamental and Interdisciplinary Sciences, Harbin Institute of Technology, Harbin 150001, China; 2 Key Laboratory of Micro-systems and Micro-structures Manufacturing, Ministry of Education, Harbin Institute of Technology, Harbin 150080, China; bguo@hit.edu.cn; 3 School of Mechatronics Engineering, Harbin Institute of Technology, Harbin 150001, China; zhangfengqiang163@163.com; 4 School of Materials Science and Engineering, Harbin Institute of Technology, Harbin 150001, China

**Keywords:** controlling distribution position of multimaterials, macroscopic manipulation, microtopography-guided substrate, deposition surface, mathematical transformation

## Abstract

Any object can be considered as a spatial distribution of atoms and molecules; in this sense, we can manufacture any object as long as the precise distribution of atoms and molecules is achieved. However, the current point-by-point methods to precisely manipulate single atoms and single molecules, such as the scanning tunneling microscope (STM), have difficulty in manipulating a large quantity of materials within an acceptable time. The macroscopic manipulation techniques, such as magnetron sputtering, molecular beam epitaxy, and evaporation, could not precisely control the distribution of materials. Herein, we take a step back and present a universal method of controlling the distribution of multimaterails during macroscopic manipulation via microtopography-guided substrates. For any given target distribution of multimaterials in a plane, the complicated lateral distribution of multimaterials was firstly transformed into a simple spatial lamellar body. Then, a deposition mathematical model was first established based on a mathematical transformation. Meanwhile, the microtopographic substrate can be fabricated according to target distribution based on the deposition mathematical model. Following this, the deposition was implemented on the substrate according to the designed sequence and thickness of each material, resulting in the formation of the deposition body on the substrate. Finally, the actual distribution was obtained on a certain section in the deposition body by removing the upside materials. The actual distribution can mimic the target one with a controllable accuracy. Furthermore, two experiments were performed to validate our method. As a result, we provide a feasible and scalable solution for controlling the distribution of multimaterials, and point out the direction of improving the position accuracy of each material. We may achieve real molecular manufacturing and nano-manufacturing if the position accuracy of distribution approaches the atomic level.

## 1. Introduction

Any object in the world can be regarded as a spatial distribution of atoms and molecules; in this sense, we can manufacture any object, if the precise distribution of atoms and molecules can be achieved. In the early 1980s, the advent of the scanning tunneling microscope (STM) made it possible to manipulate a single atom. Scientists in the International Business Machines Corporation succeeded in spelling out the “IBM” by manipulating 35 xenon atoms with STM [1,2,3,4] in 1990, declaring the coming of the era to manipulate atoms and molecules to create the world directly. Since then, to manufacture objects directly through the manipulation of atoms and molecules (e.g., molecular manufacturing) [5] has become a crucial research realm in nanotechnology. For instance, artificial quantum corrals formed by individually manipulating 48 iron atoms using STM technology can confine the electrons [6]. Multifunctional carbon nanotubes [7] and graphene [8] arranged by single carbon atoms were achieved by chemical vapor deposition. High performance heterojunctions or superlattices were realized by molecular beam epitaxy [9]. Macro-/micro- structures with a certain characteristic (e.g., adsorption [10], release [11]) were obtained by assembling atoms and molecules.

Overall, many schemes have been proposed, among which the direct manipulating and positioning of the right atoms and molecules in the right place is the most attractive one. This can be divided into two categories: one is to manipulate the atom and molecule one by one (representing point-by-point manipulation), such as STM technology [12,13,14,15,16,17,18,19,20,21,22,23]; the other is to manipulate hundreds of millions of atoms and molecules (clusters) simultaneously (representing macroscopic manipulation), such as magnetron sputtering [24,25], molecular beam epitaxy [26,27], evaporation plating [28], sublimation [29], etc. Obviously, the former can manipulate atoms and molecules precisely to the designed places to manufacture objects [12,13,14,15,16,17,18,19,20,21,22,23]; however, this is practically difficult for large-sized objects [30]. For example, to realize an object like a fly would take more than 230 million years, even at the speed of one million atoms per second [31]. In contrast, the latter can macroscopically manipulate the clusters, but fails in terms of precise position control. At this stage, we take a step back and concentrate on finding a solution to control the position of the cluster during the macroscopic manipulation. In the future, we may realize any objects, if the position accuracy of the cluster approaches the atomic or molecular level.

In this communication, we present a universal solution for controlling the position of multimaterials by using a microtopography-guided substrate. In brief, for any given target distribution of multimaterials in a plane, the complicated lateral distribution of multimaterials was first transformed into a simple spatial lamellar body. Then, a deposition mathematical model was established based on a mathematical transformation. Meanwhile, the microtopographic substrate could be fabricated according to target distribution based on the deposition mathematical model. Following this, the deposition was implemented on the substrate according to the designed sequence and thickness of each material, resulting in the formation of the deposition body on the substrate. Finally, the actual distribution was obtained on a certain section in the deposition body by removing the upside materials. The actual distribution can mimic the target one with a controllable accuracy. Furthermore, two experiments were performed to validate our method. Our method provides a feasible and scalable solution for controlling the distribution of multimaterials, and points out the direction of improving the position accuracy of each material. We may achieve real molecular manufacturing and nano-manufacturing if the position accuracy of distribution approaches the atomic level.

## 2. Experimental Details

The flat quartz substrate with a diameter and thickness of 100 mm and 3 mm, respectively, was marked on one side by direct laser writing (Figure S1a). The quartz was then ultrasonic cleaned in acetone and ethanol and deionized water for 5 min, respectively, and dried by blowing nitrogen gas. The erythrocytes were drawn from the chicken. The deposition was carried out on a high vacuum multiple target magnetron sputtering coating machine (JCP-350M2, Beijing Technol Science Co., Ltd., Beijing, China). The targets of tungsten and aluminum (99.99% purity) were purchased from Nanjing Mingshan Advanced Materials Co., Ltd. (Nanjing, China). Meanwhile, the optical images were taken from Leica DM4500P. The back scattered electron (BSE) images were taken from the field emission scanning electron microscope (SEM, Supra 55 Sapphire, Carl Zeiss, Oberkochen, Germany) at an acceleration voltage of 20 kV. The optical images were taken from Nikon D5300. The organic pigments with a size of ~1 μm were purchased from Guangdong Rainbow Deji Plastic Pigment Co., Ltd. (Guangdong, China). A series of colors (88 types) of pigments were prepared by mixing different organic pigments together. The sublimation was carried out at a temperature of 160 °C.

## 3. Results and Discussion

### 3.1. Deposition Mathematical Model

To realize a precise distribution through macroscopic manipulation, the key step is precise position control, since there are many techniques for macroscopic manipulation of the multimaterials. To this end, we have firstly introduced a mathematical transformation.

**Figure 1:**
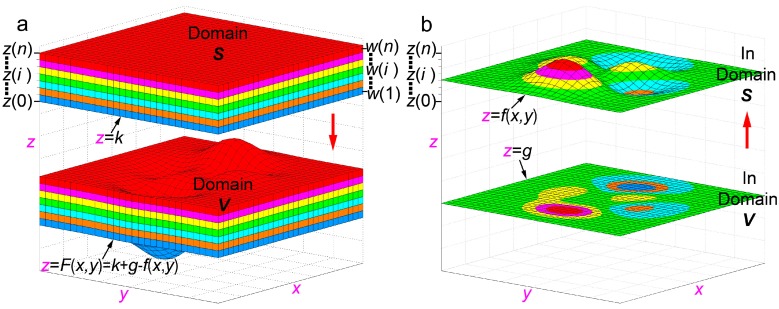
A mathematical transformation *Q*: (*x*, *y*, *z*) → (*x*, *y*, *z* + *g* − *f*(*x*, *y*)) from 3-D domain ***S*** to 3-D domain ***V***. (**a**) 3-D domain ***S*** and 3-D domain ***V***. Each color represents corresponding domain *S*(*i*) or filling material *W*(*i*). (**b**) The comparison of distribution of multimaterials both on the curved surface *z* = *f*(*x*, *y*) in domain ***S*** and that on the flat surface *z = g* in domain ***V***.

As shown in Figure 1a, we assume that *S*(*i*) is a three-dimension (3-D) domain (*a* ≤ *x* ≤ *b*, *c* ≤ *y* ≤ *d*, *z*(*i* − 1) < *z* ≤ (*i*)), (*i* = 1, 2, …, *n*), and let ***S*** = ∪ *S*(*i*). Each domain, *S*(*i*), is filled with one type of material recorded as *W*(*i*), and the thickness of *S*(*i*) (numerically equal to *W*(*i*)) is given by *h*(*i*) (*h*(*i*) = *z*(*i*) − *z*(*i* − 1)). Therefore, the domain ***S*** can be seen as a lamellar body of the same material layer *S*(*i*) (*i* = 1, 2, …, *n*).

For any given function *z* = *f*(*x*, *y*) (*a* ≤ *x* ≤ *b*, *c* ≤ *y* ≤ *d*) in the 3-D domain ***S*** (Figure 1b), and two positive numbers *k* and *g* (let *k* = *z*(0) and *k* > *g*), we establish a mathematic transformation *Q*:(*x*, *y*, *z*) → (*x*, *y*, *z* + *g* − *f*(*x*, *y*)). *V*(*i*) represents the corresponding domain of *S*(*i*) under the transformation *Q* and let ***V*** = ∪ *V*(*i*), (*i* = 1, 2, …, *n*) (Figure 1a). Thus, *Q* is a mathematical transformation from one 3-D domain ***S*** to another 3-D domain ***V*** (Figure 1a). Specifically, the *z* = *f*(*x*, *y*) in the domain ***S*** is transformed into *z* = *g* in the domain ***V*** via the transformation function *Q*, while *z* = *k* is transformed into *z* = *k* + *g* − *f*(*x*, *y*) (Figure 1a).

Following this, we define an ideal deposition for the lamellar body ***S*** of deposition materials. The ideal deposition of multimaterials *W*(1), *W*(2), …, *W*(*i*), …, *W*(*n*) is a deposition which meets the following conditions: (1) The deposition materials are points in a mathematical sense; (2) the trajectories of deposition materials are straight lines paralleled to the *z* axis; and (3) there is no secondary deposition.

Furthermore, we define the Lee Macroscopic-manipulation Precise-distribution deposition (LMP-deposition) as an ideal deposition on a curved surface, which is expressed as *z* = *F*(*x*, *y*) = *k* + g − *f*(*x*, *y*) where *z* = *f*(*x*, *y*) (*a* ≤ *x* ≤ *b*, *c* ≤ *y* ≤ *d*) is in the lamellar body ***S*** (Figure 1b), and the constants *k* and *g* meet the condition of *k* = *z*(0) > *g* > 0 (Figure 1). The curved surface is called the deposition surface, which is the core feature in the LMP-deposition. The nano-/micro- scale morphology of the deposition surface mainly has an effect on the distribution accuracy; thereby, it is a key factor for position controlling in our method.

Finally, we will discuss the relationship between the mathematical transformation *Q*: (*x*, *y*, *z*) → (*x*, *y*, *z* + *g* − *f*(*x*, *y*)) and the LMP-deposition. As shown in Figure 1a, the transformation *Q* makes the plane *z = k* in domain ***S*** transform into the curved surface *z* = *F*(*x*, *y*) = *k* + *g* − *f*(*x*, *y*) in domain ***V***. This curved surface is equal to the deposition surface in the LMP-deposition (Figure 1a). Therefore, the corresponding domain *V*(*i*) of the *S*(*i*) formed through the mathematic transformation *Q* is completely coincident with the deposition body of *V*(*i*) formed through LMP-deposition. As a result, we get an inference that for the any given surface *z* = *f*(*x*, *y*) in domain ***S***, the mathematical transformation *Q* and LMP-deposition are equivalent (Figure 1a). The inference guarantees that the conclusions drawn from the mathematical transformation *Q* can be transplanted into the LMP-deposition process. For example, the curved surface *z* = *f*(*x*, *y*) in domain ***S*** is transformed into the plane *z* = *g* in domain ***V*** through the transformation *Q*, resulting in the lateral distributions of multimaterials on both surfaces being exactly the same (Figure 1b). According to the inference, we can draw a conclusion as follows.

**Conclusion 1**: For any given curved surface *z* = *f*(*x*, *y*) in the lamellar body ***S*** of multimaterials, the lateral distribution on the section *z* = *g* in deposition body ***V*** is exactly the same as that on the surface *z* = *f*(*x*, *y*) through LMP-deposition.

### 3.2. LMP-Method

Above all, we have presented a novel method for achieving a precise position distribution of multimaterials through LMP-deposition, hereinafter referred to as the LMP-method. In the following, we have detailed the concrete steps of the LMP-method.

Step 1. Designing the target distribution of multimaterials

The domain *L* (*a* ≤ *x* ≤ *b*, *c* ≤ *y* ≤ *d*) on the target distribution is firstly divided into a number of disjoint subdomains, and each subdomain is filled with one color representing one type of material (Figure S2a bottom). Those subdomains which have the same materials are denoted as *L*(*i*), where ***L*** = ∪ *L*(*i*) (*i =* 1, 2, …, *n*), and the corresponding filled material is denoted as *W*(*i*). For example, the target distribution in Figure S2a can be divided into four colors, consisting of blue, pink, green, and red, denoting *L*(1), *L*(2), *L*(3), and *L*(4), respectively, and representing four types of deposition materials (*W*(1), *W*(2), *W*(3), *W*(4), respectively).

Step 2. Designing the sequence and thickness of deposition

**Figure 2:**
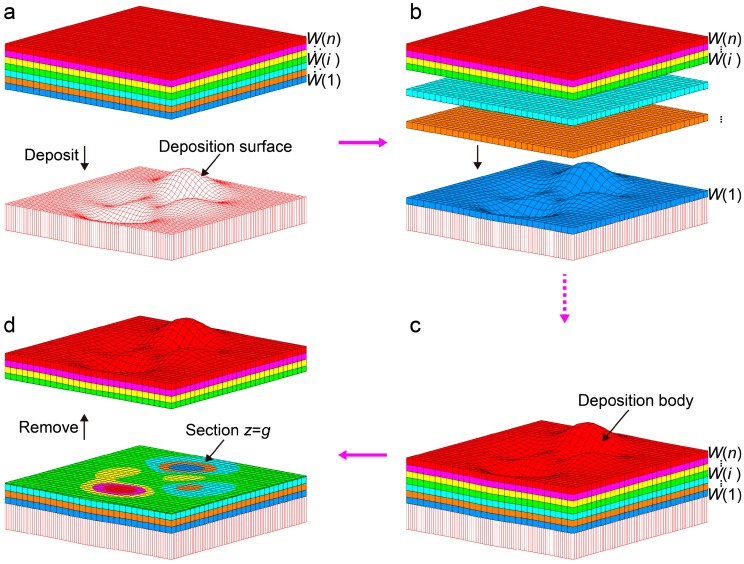
Schematic diagram of LMP-deposition model, LMP-deposition process, and actual distribution. (**a**) Schematic diagram of LMP-deposition model; (**b**,**c**) Schematic diagram of LMP-deposition process of multimaterials: the multiple materials are deposited on the deposition surface *z* = *F*(*x*, *y*) = *k* + *g* − *f*(*x*, *y*) in the sequence of *W*(1), *W*(2), …, *W*(*i*), …, *W*(*n*). Deposition direction is shown by the arrow (vertical downward). (**d**) Schematic diagram of actual distribution. Removing upside materials and getting the section *z* = *g*, which is the actual distribution of deposition materials absolutely consistent with the target distribution.

A group of positive numbers *z*(*i*) (*i* = 0, 1, 2, …, *n*) and a positive number *k* (let *k* = *z*(0)) are selected. The sequence of deposition is as follows, *W*(1), *W*(2), …, *W*(i), …, *W*(*n*), in which the deposition thickness of *W*(*i*) is equal to *z*(*i*) − *z*(*i* − 1) (*i* = 1, 2, …, *n*) (Figure 2a). In the top of Figure S2a, we set the deposition sequence as blue, pink, green, and red, and thickness (*t*) of each layer is the same (*t* = *z*(1) − *z*(0)). As a result, a lamellar body (***S***) is formed.

Step 3. Selecting the curved surface *z* = *f*(*x*, *y*) in the lamellar body ***S***

For the given target distribution on a plane (Figure 1b and Figure S2a), we can find at least one curved surface *z* = *f*(*x*, *y*) in the lamellar body ***S***, whose lateral distribution is the same as the target distribution (Figure 1b and Figure S2a). For the *L*(*i*) (*i* = 1, 2, …, *n*), the value of z*_i_* can be any which meets the inequality of *z*(*i* − 1) < *f_i_*(*x*, *y*) ≤ *z*(*i*). Obviously, the mathematical expression of the curved surface *z* = *f*(*x*, *y*) is not unique. To simplify the function, *f*(*x*, *y*) takes the same value in the same material subdomain, resulting in a step surface of the curved surface (Figure S2b). Specifically, we let *z_i_ = f_i_*(*x*, *y*) = [*z*(*i −* 1) + *z*(*i*)]/2 (*i* = 1, 2, …, *n*) in the LMP-deposition (Figure S2b). For example, for the green area *L*(3) (equal to *W*(3)) in Figure S2, the value of the curved surface *z*_3_ = *f*_3_(*x*, *y*) = [*z*(2) + *z*(3)]/2 in the domain ***S*** (Figure S2a,b). The same solution process is done for *L*(1), *L*(2), and *L*(4) in the following three layers: *W*(1), *W*(2) and *W*(4), respectively. As a result, a step surface is formed in lamellar body ***S*** (Figure S2b top).

Step 4. Preparing the deposition surface *z* = *F*(*x*, *y*) = *k* + *g* − *f*(*x*, *y*)

Since *z* = *f*(*x*, *y*) in the lamellar body ***S*** is valued in Step 3, the deposition surface can be achieved by *Q* transformation. Assuming *g* is a positive number, and *k* > *g*, the deposition surface can be determined by the function of *z* = *F*(*x*, *y*) = *k* + *g* − *f*(*x*, *y*) (Figure S2b bottom and Figure 2a). This deposition surface can be fabricated by many techniques, such as numerical control machining, additive manufacturing, and carving technology.

Step 5. Implementing of LMP-deposition

The deposition is carried out on the deposition surface via a macroscopic manipulation according to the designed sequence and thickness (Figure 2b), and the deposition body is then formed (Figure 2c and Figure S2c).

Step 6. Getting the actual distribution of multimaterials

The actual distribution is achieved on the section of *z = g* in the deposition body (Figure 2d and Figure S2d) by removing the upside materials using lathing cut (Figure S1b). Obviously, the actual distribution (Figure S2d) completely mimicks the target one (Figure S2a bottom).

**Conclusion 2**: The distribution of multimaterials obtained through the LMP-method is consistent with that in the target distribution.

### 3.3. Accuracy of LMP-Method of Manipulating Multimaterials

For the real materials distribution, we can employ the LMP-method to achieve a precise distribution by using molecular beam epitaxy, magnetron sputtering, evaporation plating, sublimation, etc. However, there is an inevitable distribution error, since the deposition materials are not the points in a mathematical sense and the actual deposition is different from the ideal deposition. The distribution error directly reflects the accuracy of the LMP-method of materials.

According to the step of the LMP-method, the accuracy of an actual distribution is mainly influenced by the following three parts, but not excluding the size of clusters, collimation error, and secondary deposition. The first one is the deposition surface error, which is determined by the machining accuracy of the substrate morphology. The second one is the lateral deposition error, which is defined as the maximum lateral error of the deposition body compared with the top plane of the prism, when deposition is processed on the top plane of the prism. The third one is the system error, which is the maximum allowable error of the deposition system. The relationship of the above three errors will be discussed further to determine the maximum value of the LMP-method.

### 3.4. Example 1: Mimic of Cell Distribution on Quartz by Using Different Rate of Tungsten/Aluminum Composites via Magnetron Sputtering

**Figure 3:**
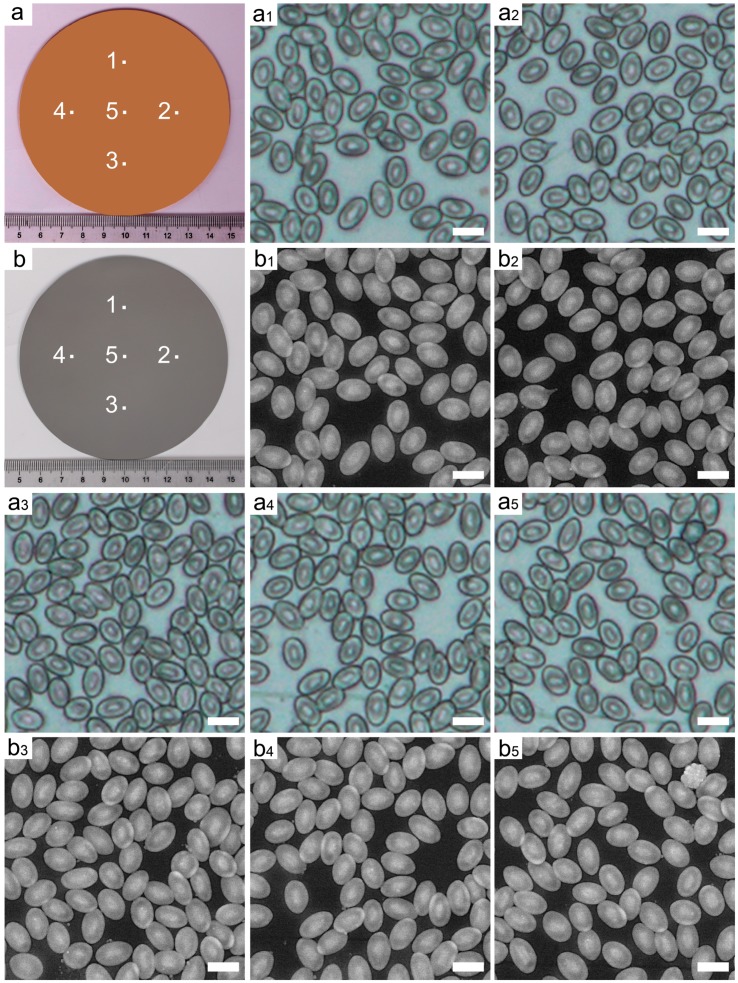
Comparison of the morphology of erythrocyte on quartz and actual distribution of different ratios of aluminum and tungsten metals. (**a**) Digital image of substrate formed by spin-coating of the erythrocytes on a quartz substrate. (**a1**–**a5**) Optical images taken on the marked areas of quartz substrate. (**b**) Digital image of the actual distribution. (**b1**–**b5**) BSE images of the actual distribution corresponding to the (**a1**–**a5**). All bars are 10 μm.

To reveal the feasibility of the LMP-method, a mimic of the cell distribution was performed. In brief, a drop of erythrocyte was spin-coated on a marked quartz substrate (Figure 3a and Figure S1a), in which the microscale morphology of erythrocytes was totally random. Optical images taken at different markers are shown in Figure 3a1–a5. Each two-dimensional (2-D) optical image can be regarded as target distribution considering that the 3D topography and its corresponding 2D image can be regarded as the different forms of the same pattern information. In addition, the 3D topography exhibits the pattern information in the form of different heights, while the 2D image represents that in the form of different colors or materials. Therefore, they are equivalent and can be transformed into each other. When the height of each point is regarded as the corresponding color (material), the topography can be transformed into the image. In contrast, when the color (material) of each point is regarded as the corresponding height, the image can be transformed into the topography. Following this, different rates of tungsten and aluminum composites as the deposition materials were deposited via the magnetron sputtering with tungsten and aluminum target in the sequence of *W*(0), *W*(1), *W*(2), *W*(*i*), …, *W*(100), in which *W*(*i*) (*i* = 0, 1, 2, …, 100) represented the atomic percentage of aluminum (*i*%) in the first *i* layer controlled by magnetron sputtering power (regulating the deposition rate of tungsten and aluminum). Each layer was set at 100 nm. Finally, the section of actual distribution (Figure 3b) was obtained by removing a certain thickness of the upside deposition body using an ultra-precision lathe machine with a high-resolution of 10 nm in dimensional accuracy (details in Figure S1b). The whole process took only 5 h. The BSE images (Figure 3b1–b5) on the actual distribution were taken from the same marked area (Figure 3a1–a5).

**Figure 4:**
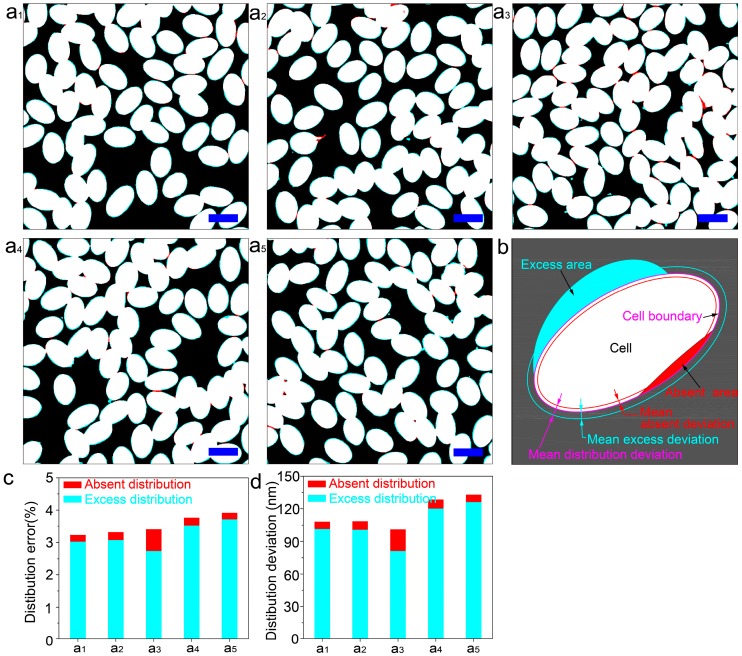
Statistical analysis of distribution error of mimic of the cell in Figure 1. (**a1–a5**) Coincidence analysis between the BSE images (Figure 1b1–b5) and the corresponding optical images (Figure 1a1–a5). The white areas show the overlapping areas between the actual distribution (BSE image) and the corresponding target distribution (optical image), in which there is no distribution error. The cyan areas around the cell represent excess areas (referring to excess distribution error) in the BSE image compared with the optical image, while the red areas at some parts of the edges of the cell represent absent areas (referring to absent distribution error). All bars are 10 μm. (**b**) Schematic diagram of distribution deviation of a single cell, in which the cell boundary, excess distribution area, absent distribution area, mean excess deviation, mean absent deviation, and mean distribution deviation are defined. (**c**) Histogram of the ratio of excess and absent distribution error according to the statistical analysis of (**a1**–**a5**). (**d**) Histogram of the mean deviation of excess and absent area according to the statistical analysis of (**a1**–**a5**).

To determine the distribution precision, coincidence analysis of the BSE images (Figure 3b1–b5) and the corresponding optical images (Figure 3a1–a5) has been conducted to get the distribution error by image processing technology using Matlab software (Figure 4). To simplify the evaluation process, both BSE and optical images were collected with the same pixels (574 × 574) and actual size (77 × 77 μm^2^) by tuning the parameters of SEM and optical equipment. As shown in Figure 4a1–a5, the white areas illustrate the overlapped areas of the BSE images and their corresponding optical images, from which the majority areas are proven to coincide very well, indicating the high accuracy of our method. To make the distribution error or accuracy clear, we defined that the excess area is the larger area in the BSE image compared to that in the optical image (Figure 4b cyan area), while the absent area is the smaller area in the BSE image compared to that in the optical image (Figure 4b red area). Accordingly, the excess distribution error can be expressed by the ratio of excess area over the whole image area, as can the absent distribution error. We assume that the total area of the cyan and red is the deviation in the actual distribution, and its ratio over the whole image (77 × 77 μm^2^) is defined as the distribution error. Furthermore, the excess area and absent area are divided by the whole length of the cell boundary (Figure 4b pink line around the cell) and forms a cyan and red line out and in the cell, respectively (Figure 4b). Accordingly, the width between the pink and cyan line is defined as the mean excess deviation, while the width between the pink and red line is defined as the mean absent deviation. The sum of the two deviation is defined as the mean distribution deviation (Figure 4b).

Figure 4c depicts the histogram of the excess and absent distribution error, from which the former is found to be much larger than the latter, suggesting a high distribution ability of our method. The final distribution error is in the range of 3.22–3.91% according to the statistical analysis (Figure 4c), which verifies the high distribution accuracy further. Meanwhile, Figure 4d depicts the histogram of mean excess and absent deviation, which shows an analogue tendency to distribution error with excess and absent deviation of 81.2–126.3 nm and 6.4–19.5 nm (Table S1), respectively. As a result, the distribution deviation of our cell example is about 100.7–132.7 nm, which is about two orders smaller than the best resolution (~15 μm) of additive manufacturing and the micromachining method [32,33,34,35]. We believe the distribution error or deviation would decrease further if the analyzed area was increased.

To reveal the component of the error, the error from the image processing should at least be considered. The boundary selection in the BSE and optical image is quite difficult owing to the very dim edges, resulting in the deviation in the calculation of the cyan and red area. We treated all the uncertain areas as the deviation, which is the up limitation of the error component induced by analysis. Therefore, the real distribution error or deviation is smaller than the above values. As a result, we can mimic an arbitrary cell distribution with a size of up to 4 inches using Al/W composites at above a 96% position accuracy (at the deviation of 132.8 nm at most) by magnetron sputtering in 5 h. This means that the morphology information of the erythrocytes can be presented by the designed materials.

### 3.5. Examples 2: Distributing Landscape Image through LMP-Method Using Sublimation Manipulation

**Figure 5:**
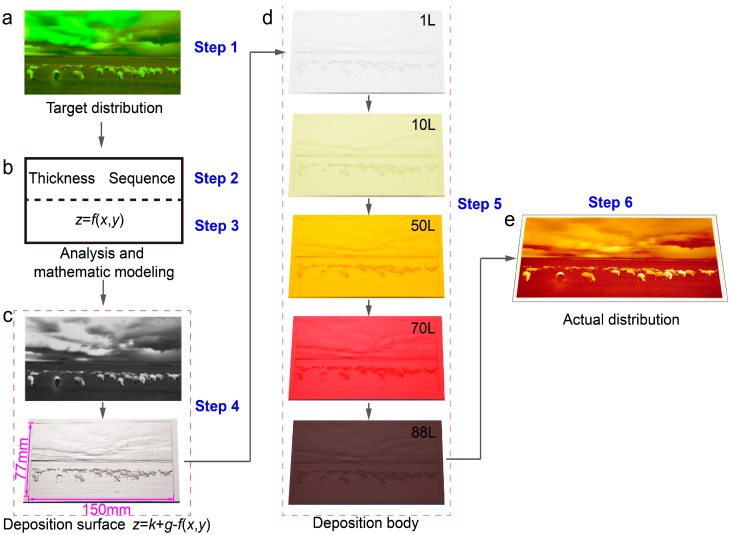
Distribution of a landscape photo via LMP-method. (**a**) Target distribution, a random digital photo of landscape with the length × width of 150 × 77 mm^2^. (**b**) Analysis and mathematic modeling to determine deposition sequence, thickness of each layer, and a function of *z* = *f*(*x*, *y*). (**c**) Fabrication of deposition surface (*z* = *k* + *g* − *f*(*x*, *y*)) via carving technology according to grayscale image of the photo. (**d**) The implement of deposition process, selected photo is the first 1, 10, 50, 70, and 88 layer, respectively. The deposition body is formed after the deposition of 88 layers. (**e**) Actual distribution obtained from a certain section (*z* = *g*) through machining the deposition body.

To reveal the generality of the LMP-method, a random digital photo of a landscape was taken as target distribution (Figure 5a). According to Figure S2, the same color in the photo was regarded as one pigment, thereby 88 types of pigments (Figure S3) were confirmed and the deposition sequence was designed based on the transformation (Figure 5b). To prepare the deposition surface, the colorful photo was firstly transferred into grayscale (Figure 5c top plan), and then drove a numerical carving machine to fabricate an anaglyph surface on the substrate. The final size of the substrate is 150 × 77 mm^2^ (Figure 5c bottom plan). The accuracy of the actual distribution was highly dependent on the carving error; nevertheless, it can decrease to a nanometer level via the current machining. The 88 layers of pigments were sublimated on the deposition surface at 160 °C in the sequence of *W*(1), *W*(2), …, *W*(*i*), …, *W*(88) (Figure 5d and Figure S3), and the deposition thickness of each layer is 10 μm. After deposition, the cross-section of the actual distribution was obtained by machining the upside deposition body (Figure S1b and Figure 5d bottom plan). Finally, the actual distribution of pigment (Figure 5e) is quite similar to the original photo (Figure 5a). The actual distribution can be unlimited close to the target through the LMP-method, if the distribution error is low enough.

### 3.6. Discussion about Materials and Techniques Dependent Constrains

The design and implementation of the LMP-method are highly dependent on many conditions, including the properties of materials, manipulating technologies, and parameters setting (such as temperature, atmosphere). There are at least two deposition modes, i.e., the physical deposition and reaction-assisted deposition. For the physical deposition, the deposited materials are just the distributed materials, while for the later, the deposited materials as intermediates evolve into the required distributed materials by a reaction with certain substances, such as a certain deposited material or atmosphere, according to the target distribution designed.

For instance, the deposition process was conducted at room temperature and in argon atmosphere to ensure physical deposition or the distribution of W/Al according to the target distribution designed in example 1. Of course, W and Al can react with each other to form intermetallics when the temperature is higher than 682 °C [36]. Also, they can be oxidized to form the corresponding oxides in the atmosphere of oxygen [37,38]. All of these properties help us to design and implement the distribution of multimaterials diversity. In example 2, the pigments are deposited at the temperature of 160 °C and in vacuum (2 × 10^−6^ tor) to ensure the corresponding deposition. Moreover, most materials, including metal, compounds, ceramics, semiconductors, and polymers, can be deposited using magnetron sputtering, molecular beam epitaxy, and thermal evaporation. This is inevitable for the diffusion of atoms during the deposition process. We may use cryogenic techniques to at least reduce the diffusion [39]. For instance, the sample holder in the magnetron sputtering is cooled by liquid helium to reduce the diffusion of materials deposited on the substrate.

Above all, for an actual application, we have to design parameters independently according to the LMP-method, such as examples 1 and 2. The LMP-method has been proven to be feasible in actual applications (e.g., examples 1 and 2) whose designs are reasonable.

## 4. Summary

In summary, we present a universal solution (i.e., LMP-method) to control the distribution of multimaterials via a microtopography-guided substrate during the macroscopic manipulation. The position of distribution materials is controlled by the microtopographic substrate and the main sources of distribution error are analyzed. Experimental results show that a quite mimicked actual distribution can be manufactured at a bulk scale in an acceptable time. Specifically, the distribution materials could be not only clusters, but also nanomaterials, supramolecular materials, macroscopic materials, and even atoms and molecules. Our method can provide a feasible and scalable solution in molecular manufacturing, nano-manufacturing, supramolecular synthesis, and micro-particles materials molding, if the size of the deposition materials decreases to a nano/micrometer level.

## Supplementary Materials

The following are available online at http://www.mdpi.com/2079-4991/8/12/1036/s1, Figure S1: (a) Marks on quartz substrate fabricated by direct laser writing and (b) Illustration of lathing to get the section of *z* = *g* in the deposition body, Figure S2: Illustration of the principle of LMP-deposition at the *z* = *f*(*x*,*y*) = [*z*(*i*) + *z*(*i* − 1)]/2, Figure S3: According to the target distribution, we have prepared 88 pigments as deposition materials, denoted as *W*(1), *W*(2)…*W*(*i*)…*W*(88), Table S1: Statistical analysis of distribution deviation in Figure 1a1–a5 images.
