# Supplementary material for: A Universal Solution of Controlling the Distribution of Multimaterials during Macroscopic Manipulation via a Microtopography-Guided Substrate
Source: Nanomaterials (Basel). 2018 Dec 12;8(12):1036. doi: 10.3390/nano8121036 (PMC6315596; doi:10.3390/nano8121036)
Supplement: Supplementary file 1 [file nanomaterials-08-01036-s001.pdf]

Supplementary Materials

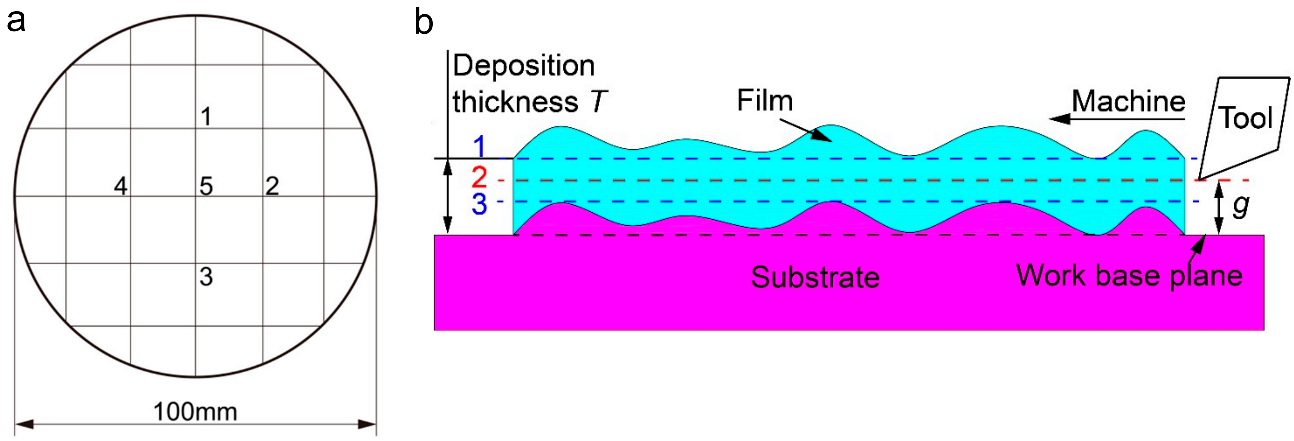

Figure S1. a) Marks on quartz substrate fabricated by direct laser writing line, the cross-link of lines are marks with number of 1, 2, 3, 4 and 5. b) Illustration of lathing to get the section of z=g in the deposition body. To get the section of z=g, the deposition body with PSS substrate was firstly clamped on the tree jaw chuck on the spindle. Then the tip of the diamond turning tool was fixed at point with distance of *g* to work base plane by CNC system. Finally, the turning process was carried out.

Table S1. Statistical analysis of distribution deviation in Figure 1a1-a5 images.

Image No.	Mean absent deviation (nm)	Mean excess deviation (nm)	Mean distribution deviation (nm)
1	6.5	101. 5	108.0
2	7.5	100.8	108.3
3	19.5	81.2	100.7
4	8.0	120.4	128.4
5	6.4	126.3	132.7

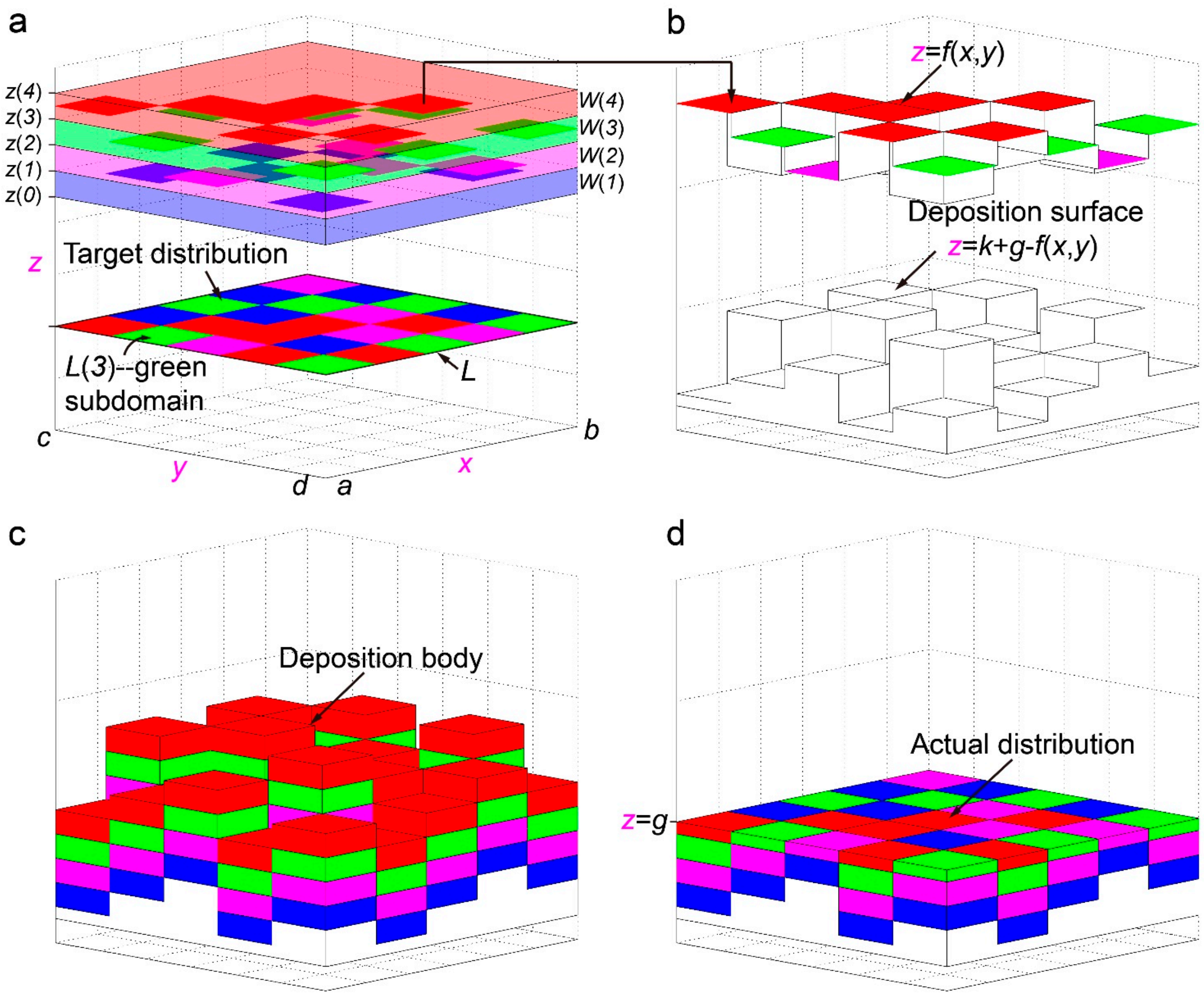

Figure S2. Illustration of the principle of LMP-deposition at the $z=f(x,y)=[z(i)+z(i\text{-}1)]/2$. a) Designing target distribution, distribution materials $W(i)$, sequence and thickness, and selecting the curved surface $z=f(x, y)$ in the lamellar body $\boldsymbol{S}$. For any point (x, y) ($a\leq x\leq b$, $c\leq y\leq d$), if the corresponding material at the point of the target distribution is $W(i)$, then $z=f(x, y)$ can be any value that meets the inequality of $z(i\text{-}1)<f\,(x, y)\leq z(i)$. To simplify calculation, we let the $f(x, y)=[z(i)+z(i\text{-}1)]/2$ in LMP-deposition. b) Preparing the deposition surface $z=F(x,y)=k+g\text{-}f(x,y)$. c) Implementing LMP-deposition and achieving deposition body. d) Getting the actual distribution of multiple materials on the section $z=g$ by removing the upside materials of deposition body

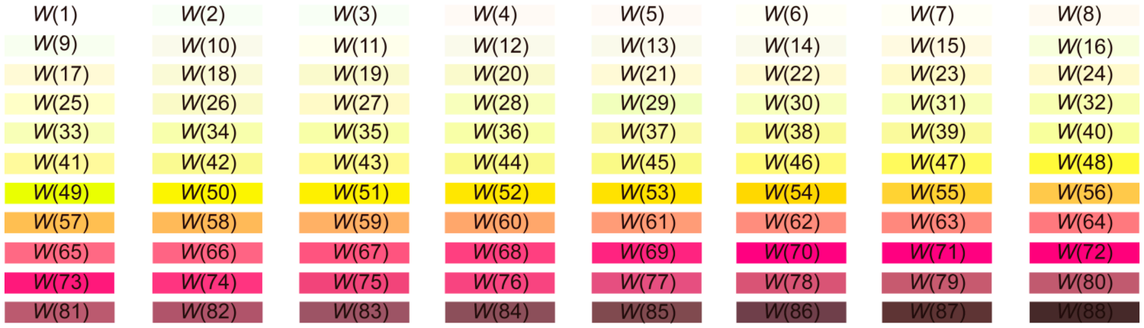

Figure S3. According to the target distribution, we have prepared 88 pigments as deposition materials, denoted as $W(1)$, $W(2)\ldots W(i)\ldots W(88)$.
